# A‐V‐V‐A response to single atrial premature depolarization in a narrow QRS tachycardia: What is the mechanism?

**DOI:** 10.1002/joa3.12924

**Published:** 2023-09-16

**Authors:** Shingo Yoshimura, Yosuke Nakatani, Kenichi Kaseno, Kohki Nakamura, Shigeto Naito

**Affiliations:** ^1^ Division of Cardiology Gunma Prefectural Cardiovascular Center Maebashi Japan

**Keywords:** atrioventricular nodal reentrant tachycardia, double ventricular response, junctional ectopic tachycardia, narrow QRS tachycardia, upper common pathway

## Abstract

We present an atypical response to single atrial premature depolarization (APD) in a long RP’ tachycardia. APD advanced the His‐bundle potential immediately after it and resulted in a VA block; however, tachycardia persisted and consequently exhibited an A‐V‐V‐A response. We propose the mechanism for an A‐V‐V‐A response to APD in a long RP' tachycardia.

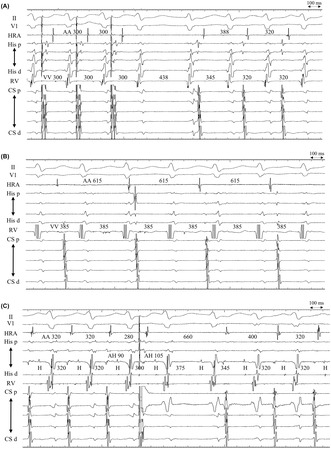

A 51‐year‐old female patient presented with repeated episodes of paroxysmal palpitations. Narrow QRS tachycardia was observed on surface electrocardiograms during palpitations. The patient underwent an electrophysiological study for narrow QRS tachycardia, and the baseline electrocardiogram showed sinus rhythm without evidence of pre‐excitation. The Atrio‐Hisian (AH) and His‐ventricular intervals were 131 and 42 ms, respectively. Two discrete discontinuities were observed in the AH conduction curve during programmed atrial extrastimulation, suggesting triple antegrade atrioventricular (AV) nodal pathways. Programmed ventricular stimulation revealed ventriculoatrial (VA) conduction with a long VA interval and decremental conduction property. Long RP’ tachycardia was spontaneously initiated without an AH jump‐up during isoproterenol infusion. Additionally, the atrial potential sequence during tachycardia was concentric and identical to that during ventricular stimulation. After cessation of atrial overdrive pacing (AOP), tachycardia exhibited an A‐V‐V‐A response (Figure 1A). The tachycardia persisted intermittently with VA dissociation (Figure 1B). Ventricular premature depolarization (VPD) delivered during the refractory period of the His‐bundle failed to reset tachycardia. Ventricular overdrive pacing during tachycardia could not retrogradely capture the atrium. The responses to atrial premature depolarization (APD) delivered during tachycardia are shown in Figure 1C.

**FIGURE 1:**
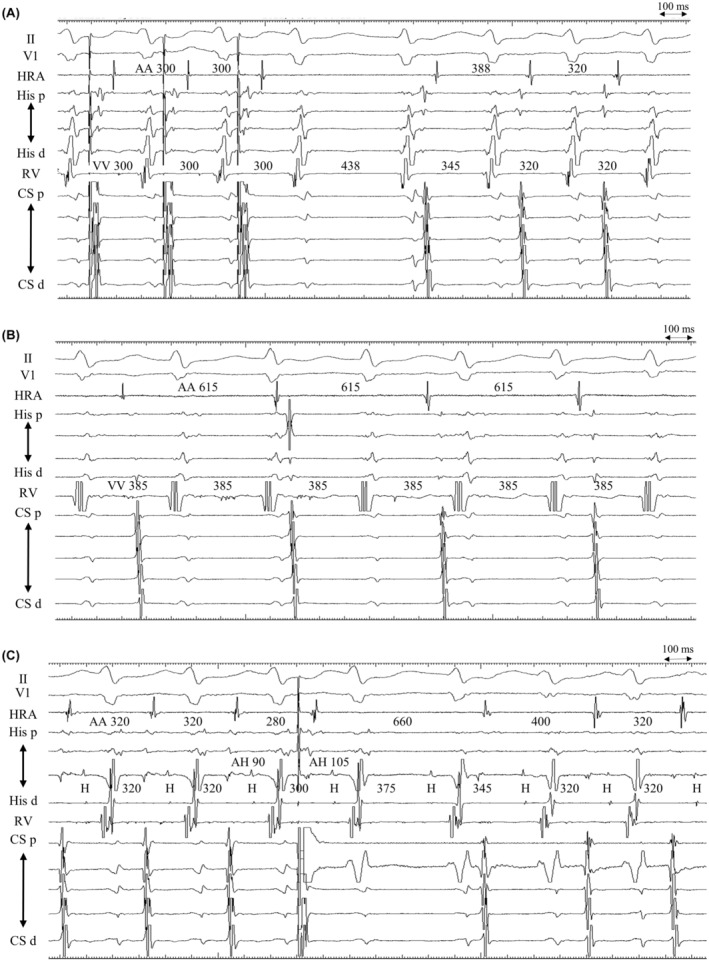
(A) An A‐V‐V‐A response after the cessation of atrial overdrive pacing at a pacing cycle length of 300 ms. (B) Tachycardia persisted with ventriculoatrial dissociation. The sequence of atrial potentials was identical to that during sinus rhythm, with a cycle length of 615 ms. (C) Responses to atrial premature depolarizations during tachycardia with coupling interval of 280 ms. II, V1, surface electrogram; CS d, distal coronary sinus; CS p, proximal coronary sinus; His d, distal His‐bundle region; His p, proximal His‐bundle region; HRA, high right atrium; RV, right ventricle.

The differential diagnoses of narrow QRS tachycardia are as follows: atypical atrioventricular nodal reentrant tachycardia (AVNRT), junctional ectopic tachycardia (JET), atrial tachycardia, and orthodromic reciprocating tachycardia (ORT) using a slowly conducting AV accessory or a concealed nodoventricular/nodofascicular bypass tract. Atrial tachycardia and ORT using the AV accessory bypass tract were ruled out by VA dissociation (Figure 1B) because the atrium was necessary for the tachycardia. ORT using a concealed nodoventricular/nodofascicular bypass tract was also unlikely because VPD delivered from the right ventricle during the His refractory period did not reset the tachycardia.^1^

The possible diagnoses were AVNRT and JET. APD during tachycardia is one of the maneuvers to differentiate between AVNRT and JET.^2^ Although APD delivered during His‐bundle refractoriness cannot reset tachycardia in JET, it can advance or delay tachycardia in AVNRT. In this case, APD advanced the His‐bundle potential immediately after it by 20 ms and resulted in a VA block. The His‐bundle potential after the VA block was delayed by 55 ms. Consequently, tachycardia exhibited an A‐V‐V‐A response (Figure 1C). The interval between the His‐bundle potential before and one beat after APD was significantly longer than that of two beats of tachycardia cycle length (300 + 375 ms > 320 ms × 2). This finding is unlikely in JET, therefore supporting the diagnosis of AVNRT. The AVNRT should have used a fast pathway (FP) as the anterograde limb, and a slow pathway (SP1) as the retrograde limb, since tachycardia was initiated without the AH jump‐up and the long RP’ tachycardia. Since tachycardia persisted with VA dissociation, it was diagnosed as a fast‐slow AVNRT with an upper common pathway.^3^

The A‐V‐V‐A response after APD can be explained by an upper common pathway block. We estimated the effective refractory period of the upper common pathway to be approximately 280–290 ms. The wavefront from the APD propagated anterogradely over the FP, encroaching on the retrograde SP1. Retrograde SP1 conduction failed to reach the atrium due to the refractory period of the upper common pathway; however, conduction proceeded anterogradely to the FP, sustaining tachycardia. Retrograde SP1 conduction after the VA block proceeded through the upper common pathway owing to recovery of the refractoriness (Figure 2B).

**FIGURE 2:**
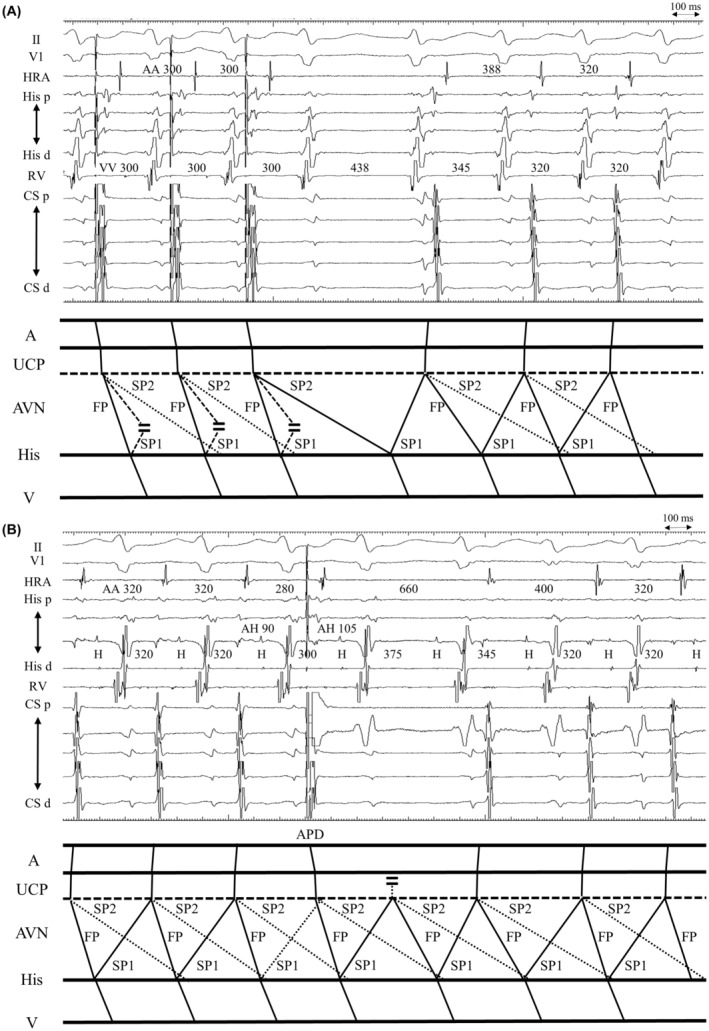
The tracing is shown in Figure 1A,C and its corresponding laddergram. II, V1, surface electrogram; A, atrium; APD, atrial premature depolarizations; AVN, atrioventricular node; CS d, distal coronary sinus; CS p, proximal coronary sinus; FP, fast pathway; His d, distal His‐bundle region; His p, proximal His‐bundle region; HRA, high right atrium; RV, right ventricle; SP, slow pathway; UCP, upper common pathway; V, ventricle.

A previous study showed AOP during tachycardia as another maneuver for distinguishing AVNRT from JET.^4^ An A‐V‐V‐A response after the cessation of AOP favors JET as a mechanism (Figure 1A). However, this mechanism can also be explained by a double ventricular response. Activation from the last beat of the AOP propagated simultaneously through FP, SP1, and another slow pathway (SP2). Antegrade conduction of the FP propagated the retrograde SP1, which was blocked due to collision with antegrade SP1. Although the AOP terminated the tachycardia, antegrade SP2 from its last beat proceeded to a retrograde SP1, subsequently reinitiating the tachycardia (Figure 2A).

Mapping atrial activation during tachycardia revealed that the earliest site was close to the His bundle. Therefore, this finding suggests that the upper common pathway was connected to the atrium of the His bundle area. Considering the risk of AV block by ablation due to the possibility of proximity between FP and SP1, we did not ablate the SP1 and opted for drug therapy.

To the best of our knowledge, this is the first case report of an A‐V‐V‐A response to APD in a fast‐slow AVNRT.

## CONFLICT OF INTEREST STATEMENT

Authors declare no conflict of interests for this article.

## INFORMED CONSENT

Informed consent was obtained from the patient.
